# Treatment of out-of-hospital cardiac arrest in the COVID-19 era: A 100 days experience from the Lombardy region

**DOI:** 10.1371/journal.pone.0241028

**Published:** 2020-10-22

**Authors:** Enrico Baldi, Giuseppe Maria Sechi, Claudio Mare, Fabrizio Canevari, Antonella Brancaglione, Roberto Primi, Alessandra Palo, Enrico Contri, Vincenza Ronchi, Giorgio Beretta, Francesca Reali, Pier Paolo Parogni, Fabio Facchin, Ugo Rizzi, Daniele Bussi, Simone Ruggeri, Luigi Oltrona Visconti, Simone Savastano, all the Lombardia CARe researchers

**Affiliations:** 1 Department of Molecular Medicine, Section of Cardiology, University of Pavia, Pavia, Italy; 2 Cardiac Intensive Care Unit, Arrhythmia and Electrophysiology and Experimental Cardiology, Fondazione IRCCS Policlinico San Matteo, Pavia, Italy; 3 Azienda Regionale Emergenza Urgenza, Milano, Italy; 4 SOREU della Pianura, Azienda Regionale Emergenza Urgenza (AREU), Pavia, Italy; 5 Division of Cardiology, Fondazione IRCCS Policlinico San Matteo, Pavia, Italy; 6 AAT Pavia—Azienda Regionale Emergenza Urgenza (AREU) c/o Fondazione IRCCS Policlinico San Matteo, Pavia, Italy; 7 AAT Pavia—Azienda Regionale Emergenza Urgenza (AREU) c/o ASST di Pavia, Pavia, Italy; 8 AAT Lodi—Azienda Regionale Emergenza Urgenza (AREU) c/o ASST di Lodi, Lodi, Italy; 9 AAT Mantova—Azienda Regionale Emergenza Urgenza (AREU) c/o ASST di Mantova, Mantova, Italy; 10 AAT Cremona—Azienda Regionale Emergenza Urgenza (AREU) c/o ASST di Cremona, Cremona, Italy; University of Palermo, ITALY

## Abstract

**Introduction:**

An increase in the incidence of OHCA during the COVID-19 pandemic has been recently demonstrated. However, there are no data about how the COVID-19 epidemic influenced the treatment of OHCA victims.

**Methods:**

We performed an analysis of the Lombardia Cardiac Arrest Registry comparing all the OHCAs occurred in the Provinces of Lodi, Cremona, Pavia and Mantua (northern Italy) in the first 100 days of the epidemic with those occurred in the same period in 2019.

**Results:**

The OHCAs occurred were 694 in 2020 and 520 in 2019. Bystander cardiopulmonary resuscitation (CPR) rate was lower in 2020 (20% vs 31%, p<0.001), whilst the rate of bystander automated external defibrillator (AED) use was similar (2% vs 4%, p = 0.11). Resuscitation was attempted by EMS in 64.5% of patients in 2020 and in 72% in 2019, whereof 45% in 2020 and 64% in 2019 received ALS. At univariable analysis, the presence of suspected/confirmed COVID-19 was not a predictor of resuscitation attempt. Age, unwitnessed status, non-shockable presenting rhythm, absence of bystander CPR and EMS arrival time were independent predictors of ALS attempt. No difference regarding resuscitation duration, epinephrine and amiodarone administration, and mechanical compression device use were highlighted. The return of spontaneous circulation (ROSC) rate at hospital admission was lower in the general population in 2020 [11% vs 20%, p = 0.001], but was similar in patients with ALS initiated [19% vs 26%, p = 0.15]. Suspected/confirmed COVID-19 was not a predictor of ROSC at hospital admission.

**Conclusion:**

Compared to 2019, during the 2020 COVID-19 outbreak we observed a lower attitude of laypeople to start CPR, while resuscitation attempts by BLS and ALS staff were not influenced by suspected/confirmed infection, even at univariable analysis.

## Introduction

A prompt treatment grounded in basic and advanced interventions is essential for out-of-hospital cardiac arrest (OHCA) victims’ survival. During COronaVIrus Disease 19 (COVID-19) pandemic, which emerged in China at the end of December 2019 [1], an impressive increase of OHCAs has been documented [2, 3] with a different magnitude according to the incidence of COVID-19 case in the different regions. It has been also shown that the trend of the epidemic (i.e. the evolution of the incidence of COVID-19 cases in a region) was significantly correlated with the excess of OHCA in 2020 suggesting many different causative mechanisms mainly ascribable either to the infection-related or to the pandemic-related issues [4]. These factors led to consider the COVID-19 epidemic as a major challenge for rescue in the context of OHCA, especially considering the complex scenario for both patients and rescuers. This complexity was the outgrowth of many participating factors such as the overload of the emergency system, the increase of the infective risk for the rescuers at different level and the perceived futility of the resuscitation maneuvers for COVID-19 patients. As a matter of fact, experts [5] and preliminary data [6] suggested a poor outcome of COVID-19 patients who suffered a cardiac arrest. Moreover, rescuers are called to face a different kind of resuscitation burdened by the discomfort [7] and the time need to wear the Personal Protection Equipment (PPE), essential to face the increased infective risk. For all the above, the major scientific societies and the International Liaison Committee on Resuscitation (ILCOR) released specific guidelines and recommendations both for the basic and the advanced treatment of cardiac arrest victims [8–10]. Our aim was to assess how the COVID-19 epidemic influenced the treatment of OHCA victims in the Lombardy region, one of the first affected regions outside China [11], with the first case diagnosed on February 20^th^, 2020 in Lodi Province.

## Materials and methods

### Study population

Lombardia Cardiac Arrest Registry (Lombardia CARe—NCT03197142) is a multicenter longitudinal prospective registry that has been enrolling all the OHCAs from the Province of Pavia since January 2015 and from the Provinces of Lodi, Cremona, Mantua and Pavia since January 2019. All the data are collected following Utstein 2014 recommendations [12]. It was approved by the Ethical Committee of the Fondazione IRCSS Policlinico San Matteo (proc. 20140028219) and by all the others involved in the territory.

We considered all the OHCAs that occurred in these four Provinces in the southern part of the Lombardy Region, in northern Italy, in the first 100 days of epidemic following the first documented case in the Lombardy Region (February 21^st^, 2020 to May 30^th^, 2020) and those of the same time frame in 2019 (February 21^st^, 2019 to May 31^st^, 2019, to account for the leap year).

All the Emergency Medical System (EMS) electronic records have been reanalyzed. We computed the number of patients with symptoms compatible with COVID-19 (fever for at least three days before OHCA associated with cough and/or dyspnea), who were considered as suspected for COVID-19 as the COVID-19 cannot be ruled out, and the number of patients with confirmed COVID-19 (diagnostic pharyngeal swab).

### Data management

The data of the study are collected and managed using REDCap electronic data capture tools hosted at Fondazione IRCCS Policlinico San Matteo [13, 14]. REDCap (Research Electronic Data Capture) is a secure, web-based software platform designed to support data capture for research studies, providing 1) an intuitive interface for validated data capture; 2) audit trails for tracking data manipulation and export procedures; 3) automated export procedures for seamless data downloads to common statistical packages; and 4) procedures for data integration and interoperability with external sources.

### Statistical analysis

Categorical variables were compared with the Chi-square test and presented as number and percentage. Continuous variables were compared with the t-test and presented as mean ± standard deviation or compared with the Mann-Whitney test and presented as median and 25–75 interquartile range (IQR) according to normal distribution tested with the D’Agostino-Pearson test. Univariable and multivariable logistic regression models were built to test for independent predictors of ALS started. In the multivariable model only non-correlated variables and statistically significant predictors at univariable analysis were inserted. Statistical analyses were performed with the MedCalc version 19.2 (MedCalc Software Ltd). A p-value less than 0.05 was considered statistically significant.

### Setting and Emergency Medical System (EMS) description

The total area covered by the Lombardia CARe registry is of 7,863‬ km^2^ divided into the four provinces (Pavia 2,969 km^2^; Lodi 783 km^2^; Cremona 1,770 km^2^; Mantova 2,341 km^2^). Each province has several rural regions and a few urban areas for a total population of 1,545,849 inhabitants (Pavia 545,810; Lodi 229,765; Cremona 358,512; Mantova 411,762) as of January 1st, 2019 and 1,547,333 (Pavia 545,888; Lodi 230,198; Cremona 358,955; Mantova 412,292) as of January 1st, 2020. The EMS dispatch center is unique for all the four provinces and coordinates 45 ambulances staffed with basic life support—defibrillation (BLS-D)-trained personnel, and 21 advanced life support (ALS)-trained staffed vehicles (a physician and a specialized nurse or a specialized nurse only). Five helicopters with a physician and a specialized nurse on board also serve the entire region of Lombardy and other three can intervene from other neighboring regions. In case of suspected OHCA, the EMS dispatcher activates one to three emergency vehicles (which may include a helicopter) with at least one physician and assists the calling bystander during chest compressions (telephone CPR with chest-compression only technique). The decision about the attempt and the duration of resuscitation are left to the physician, whilst BLS-D-trained personnel are instructed to start resuscitation unless clear signs of death are present (rigor mortis, hypostasis, and injuries not compatible with life).

### Emergency Medical System (EMS) adaptation to the COVID-19 outbreak

During the COVID-19 outbreak, the number of BLS-D and ALS trained staffed vehicles increased by 40% (from 45 to 63) and by 29% (from 21 to 27), respectively. This increase was achieved thanks to the extraordinary effort of volunteer staff for BLS-D-trained staffed vehicles and the increase in shifts of physician and nurses for ALS-trained staffed vehicles. The EMS dispatchers and personnel were instructed to investigate for any possible symptoms related to COVID-19, both during the emergency call and at the patient’s side from the caller, the relatives and the eventual bystanders. Moreover, the EMS personnel had been instructed to wear personal protective equipment (face shield, N95 face mask, isolation gown) before starting resuscitation in case of doubt for or diagnosed of COVID-19. No specific changes in BLS or ALS protocols were adopted during the COVID-19 epidemic.

## Results

The OHCAs occurred were 1214 in the study period (694 in 2020 and 520 in 2019).

The OHCAs occurred in 2020 and 2019 were similar concerning age and gender, whereas the medical etiology and the home location were prevalent in 2020 compared to 2019. Moreover, a non-significant trend towards a reduction in shockable presenting rhythm was observed in 2020 and the EMS arrival time was 3-minutes longer in 2020 compared to 2019 (Table 1). The number of patients presented with symptoms suspected for COVID-19 before the OHCA or a diagnosis of COVID-19 before or after the OHCA was 139 and their characteristics are presented in S1 Table.

**Table 1:** Baseline characteristics of OHCA occurred in 2020 and in 2019.

*Variable*	*2020*	*2019*	*P*
	*n = 694*	*n = 520*	
**Males, n (%)**	430 (62)	300 (57.7)	0.28
**Age, years [IQR]**	77 [67–85]	79 [65–86]	0.14
**Suspected or confirmed COVID-19 infection, n (%)**	139 (20)	0 (0)	-
**EMS arrival time, mins [IQR]**	15 [11–19]	12 [9–15]	<0.001
**Etiologies, n (%)**			0.002
*Medical*	613 (93)	456 (89)	
*Trauma*	23 (3.5)	44 (8.6)	
*Drowning*	1 (0.2)	0 (0)	
*Overdose*	4 (0.6)	1 (0.4)	
*Electrocution*	0 (0)	0 (0)	
*Asphyxial (external causes)*	21 (3.2)	10 (2)	
*Unknown*	32 (4.6)	9 (1.7)	
**OHCA locations, n (%)**			
*Home*	623 (90)	420 (81)	<0.001
*Nursing residence*	33 (4.8)	37 (7.1)	
*Workplace*	3 (0.4)	4 (0.8)	
*Street*	26 (3.7)	44 (8.5)	
*Public building*	4 (0.6)	9 (1.7)	
*Sport*	0 (0)	2 (0.4)	
*Other*	5 (0.7)	4 (0.8)	
**Witnessed status, n (%)**			0.002
*Unwitnessed*	344 (50)	219 (42)	
*Bystander witnessed*	236 (34)	235 (45)	
*Witnessed by EMS*	86 (12)	55 (11)	
*Unknown*	28 (4)	11 (2)	
**Shockable presenting rhythm, n (%)**	56 (8)	58 (11)	0.07
	56 (12.5)^¥^	58 (15.5)^¥^	0.2

IQR: 25–75 percentile range

EMS: Emergency Medical System

OHCA: out-of-hospital cardiac arrest

CPR: cardiopulmonary resuscitation

ROSC: return of spontaneous circulation

¥ considering only patients with resuscitation attempted

Concerning bystander intervention, the rate of bystander cardiopulmonary resuscitation (CPR) in 2020 was significantly lower than in 2019 (24.5% vs 35.7%, p<0.001), but the rate of use of an automated external defibrillator (AED) by bystander was similar (2% vs 4%, p = 0.11) (Table 2).

**Table 2:** Bystanders and EMS intervention and resulting short-term outcome.

Variable	2020	2019	p
	n = 694	n = 520	
Bystanders resuscitation			
Bystander CPR, n (%)	140 (24.5)^	162 (35.7)^	<0.001
	126 (34.6)†	154 (48.3)†	<0.001
AED use before EMS Arrival, n (%)	13 (2)	20 (4)	0.11
**EMS Resuscitation**			
Resuscitation attempted, n (%)	448 (64.5)	373 (72)	0.008
Cause for not resuscitation, n (%)			0.07
*obviously dead*	204 (29.5)	111 (21)	
*considered futile*	42 (6)	36 (7)	
ALS attempted, n (%)	200 (29)	239 (46)	<0.001
	200 (45)^¥^	239 (64)^¥^	<0.001
Mechanical compression, n (%)	27 (13.5)*	37 (15.5)*	0.6
Epinephrine, mg [IQR]	4 [2–5]*	3 [2–5]*	0.15
Amiodarone, n (%)	28 (14)*	28 (12)*	0.3
Shock delivered (mean±SD)	1.2±2.3*	1.15±2.6*	0.87
Resuscitation duration median^¶^ [IQR]	21.6 [10.3–36.4]	23.1 [12.5–36.3]	0.3
**Outcome**			
ROSC at hospital admission, n (%)	48 (11) ^¥^	73 (20) ^¥^	0.001
	37 (19)*	61 (26)*	0.15

CPR Cardiopulmonary resuscitation.

AED Automated external defibrillator.

ALS Advanced Life Support.

IQR 25–75 Interquartile range.

ROSC Return Of Spontaneous Circulation.

^ excluding EMS-witnessed patients.

† excluding EMS-witnessed patients and considering only patients with resuscitation attempted.

¥ considering only patients with resuscitation attempted.

* considering only patients with ALS attempted.

¶ intended as the time from EMS arrival to the end of resuscitation.

Resuscitation was attempted by EMS in 448 (64.5%) patients in 2020 and in 373 (72%) in 2019, whereof 200 (45%) in 2020 and 239 (64%) in 2019 received ALS (Table 2). At univariable analysis, the presence of suspected or confirmed COVID-19 was not a predictor of resuscitation attempt [OR 1.3 (0.9–2), p = 0.17].

The percentage of males, medical etiology, EMS-witnessed, bystander-witnessed, public location of OHCA, shockable presenting rhythm, bystander CPR, resuscitation attempted, ALS attempted and ROSC at hospital admission in different groups of patients are presented in S1 Fig.

**Fig 1:**
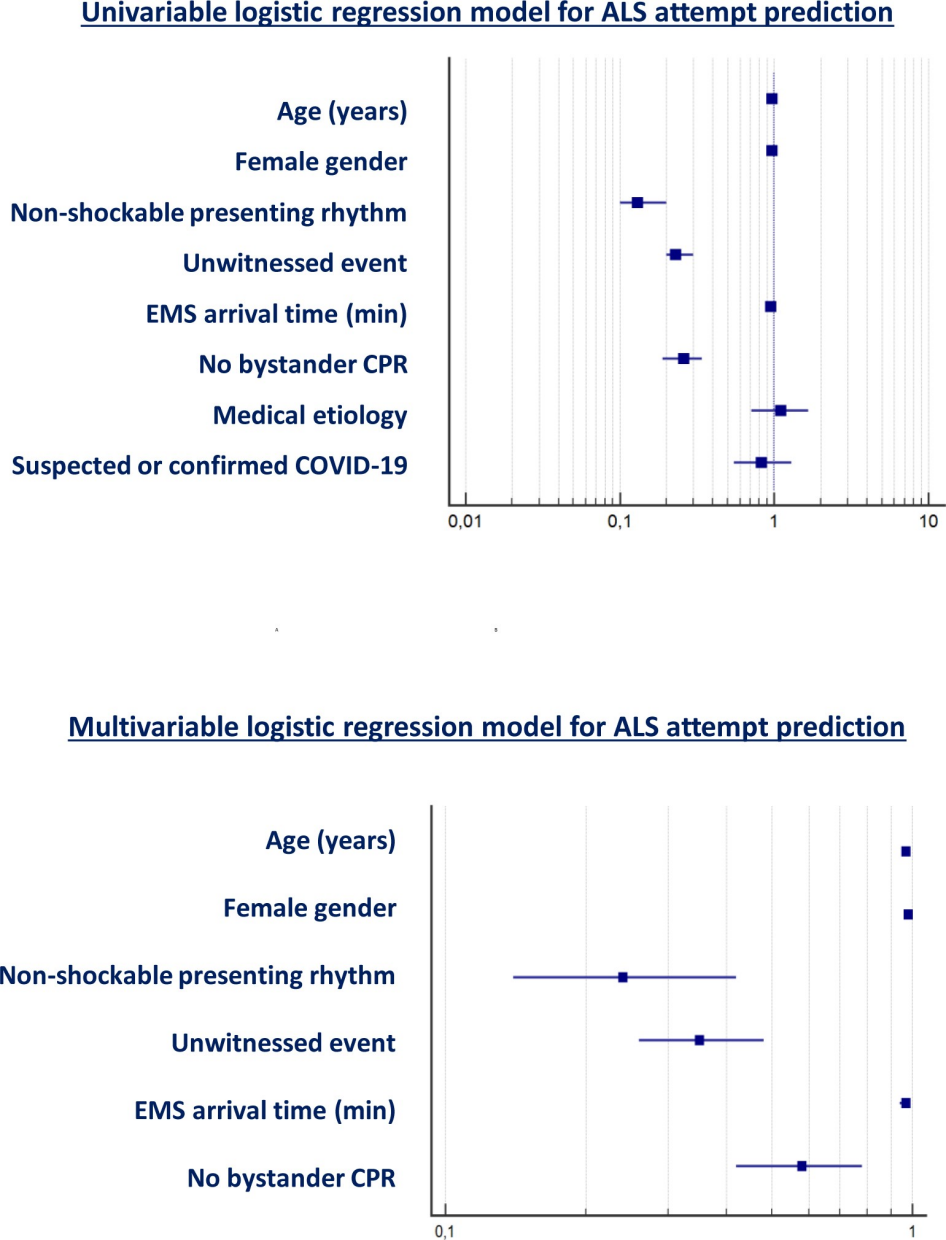
Forest plot for univariable and multivariable logistic regression model for ALS attempt prediction.

Concerning advanced resuscitation, the age, the gender, a non-shockable presenting rhythm, the unwitnessed status, the EMS arrival time and the absence of bystander CPR were predictors of ALS attempt at univariable analysis; on the contrary, the medical etiology and the presence of a suspected or confirmed COVID-19 infection were not. At multivariable analysis, the age, the unwitnessed status, the non-shockable presenting rhythm, the absence of bystander CPR and the EMS arrival time were confirmed to be independent predictors of ALS attempt (Table 3 and Fig 1).

**Table 3:** Univariable and multivariable logistic regression model for ALS attempt prediction.

	Univariable logistic regression for ALS attempt			Multivariable logistic regression for ALS attempt			
Variable	OR	95%CI	p	OR	95%CI	Wald	p
**Age (years)**	0.97	0.97–0.98	<0.001	0.97	0.96–0.98	43.6	<0.001
**Female gender**	0.97	0.95–0.99	0.02	0.98	0.96–1	1.8	0.18
**Non-shockable presenting rhythm**	*0*.*13*	*0*.*1–0*.*2*	*<0*.*001*	0.24	0.14–0.42	26.6	<0.001
**Unwitnessed event**	0.23	0.2–0.3	<0.001	0.35	0.26–0.48	44.6	<0.001
**EMS arrival time (min)**	0.95	0.94–0.97	<0.001	0.97	0.94–0.99	9.2	0.002
**No bystander CPR**	0.26	0.19–0.34	<0.001	0.58	0.42–0.78	11.4	0.001
**Medical etiology**	1.1	0.71–1.66	0.69				
**Suspected or confirmed COVID-19 infection**	0.83	0.55–1.3	0.4				

Considering only those patients who received ALS (200 in 2020 and 239 in 2019), we did not find significant difference in term of treatment comparing the two years. The duration of resuscitation [21.6 (10.3–36.4) min vs 23.1 (12.5–36.3) min, p = 0.3], the total amount of epinephrine administered [4 (2–5) mg vs 3 (2–5) mg, p = 0.15], the rate of patients receiving amiodarone [28 (14%) vs 28 (12%), p = 0.3], the number of shocks delivered [1.2±2.3 vs1.15±2.6, p = 0.87] and the use of a mechanical device for chest compressions [27 (13.5%) vs 37 (15.5%), p = 0.6] were indeed similar between the two years (Table 2).

As far as the short-term outcome is concerned a significantly reduction of the rate of return of spontaneous circulation (ROSC) at hospital admission was observed in the general population [48 (11%) in 2020 vs 73 (20%) in 2019, p = 0.001], but, when considering only the patients with ALS initiated, we pointed out a similar rate of ROSC at hospital admission [37 (19%) in 2020 vs 61 (26%) in 2019, p = 0.15] (Table 2). Interestingly, in the general population, the presence of suspected or confirmed COVID-19 was not an independent predictor of ROSC at hospital admission [0.9 (0.4–2.1), p = 0.78] after correction for age, gender, presenting rhythm, witnessed status, bystander CPR and EMS arrival time.

## Discussion

We present the largest series regarding OHCA treatment during COVID-19 pandemic from one of the first burned areas worldwide such as the Lombardy region. There are several demonstration that the COVID-19 pandemic led to an increase in mortality [15] and OHCA incidence [2, 3, 16], whilst no data are available so far regarding the treatment of these patients. Our data firstly demonstrate that ALS treatment is essential for OHCA survival also in COVID-19 era and that the presence of COVID-19 diagnosis or symptoms is not a predictor of ROSC.

Many of our results are worthy of discussion. The first is that the characteristics of OHCAs were quite different in the COVID-19 era when compared to the same period of the previous year, with a predominance of both home location and medical etiology. This finding confirmed our previous results [2, 4] and those observed in France [3] and it is fully consistent with both the quarantine constriction and the infection related causes of cardiac arrest [4]. We also observed a non-significant trend towards a reduction in shockable presenting rhythm. Many aspects may have influenced the presenting rhythm: an increase in primarily hypoxic OHCA due to COVID-19 [4] and the 3-minutes increase in EMS arrival time may have led to an increase in not-shockable rhythm [17], whilst the observed reduction in patients referring to the EMS for myocardial infarction and therefore the increase in cardiovascular complication [18, 19] leading to OHCA in these patients may have increased the shockable ones. Moreover, in 2020 there was a significantly reduction of bystander CPR. Three reasons seem possible: the first is that during pandemic, probably because of lockdown and social distancing, there was an increase in home location of the OHCAs, which is known to be associated with a lower rate of bystander CPR [20]; the second is that the fear of infection was one of the main concern of lay rescuers also before COVID-19 pandemic [21, 22], and if it could limit the rate of bystander intervention in normal conditions, it is by far likely to be the main driver of this reduction during this outbreak; the third is that also in OHCAs occurred in the public place, the probability of the presence of a witness and to receive CPR was lower due to the limited presence of people outside the houses.

Another element worthy of discussion is that if on one side the fear of infection probably influenced lay rescuers, it appeared neutral on EMS rescuers. Supporting this, we found that the presence of a suspected or confirmed COVID-19 infection was not a predictor of resuscitation or ALS attempt. This means that both BLS-D staffed and ALS staffed EMS personnel were not influenced neither by the fear of infection, probably mitigated by the use of adequate PPE, nor by the thinking of a possible futile resuscitation in such a case. The decision to perform advanced resuscitation seems to be based on OHCAs’ characteristics, as normally occurs. Age, gender, presenting rhythm, witnessed event, EMS arrival time and bystander CPR, which were found to be independent predictors of EMS resuscitation attempt, were indeed already demonstrated to be associated to EMS decision to initiate CPR some years ago [23, 24]. However, during COVID-19 pandemic, there was an increase in non-witnessed OHCAs with less bystander CPR and longer EMS arrival time, and all these factors, related to a direct or an indirect effect of the pandemic disease, led to a decrease in resuscitation attempted by EMS.

Another point of discussion is that when ALS was started by EMS, it was performed similarly to what happened in 2019, outside the epidemic. There were indeed no difference in the duration of resuscitation attempts, in the milligrams of epinephrine administered, in the administration rate of amiodarone, in the use of mechanical compression device and in the number of shocks delivered. This was not obvious as it was shown that the wearing of PPE could limit the execution of advanced resuscitation maneuvers [7]. On the contrary, in our experience, the duration of resuscitation and the kind of interventions were similar to 2019 indicating that also in this particular scenario rescuers made the same life-saving ALS effort.

Finally, concerning the short-term outcome, the rate of ROSC was lower in 2020 in the general population of OHCAs and this is consistent also with data presented by Marijon et al. regarding the Parisian experience, where the survival to hospital admission decrease from 22.8% to 12.9% during pandemic period, very similarly as in our region [3]. However, we highlighted that the rate of ROSC at hospital admission was similar to the previous year when considering only those patients who received ALS. This is an important result and it indicates, alongside to the issues discussed above, the need of a proper patients’ selection and the importance to initiate ALS-treatment as normally done during a cardiac arrest rescue prior to the COVID-19 epidemic. Moreover, it could be expected that the presence of a suspected or confirmed COVID-19 could led to a worst prognosis. Conversely, we found that it was not an independent predictor of ROSC suggesting the possible use of validated scores [25, 26] to estimate the probability of survival also during the epidemic.

## Limitations

The first limitation is that data come from an observational registry, but we think this is the only weapon to have real-life data in such a particular situation as an epidemic.

The second limitation is that we do not have data about both the airways management and the route for drug administration. As stated in the methods the Lombardia CARe is an Utstein based registry and according the 2014 Utstein guidelines both these data are not considered mandatory.

## Conclusions

In the largest series concerning the treatment of out-of-hospital cardiac arrest in the COVID-19 era, we observed a lower attitude of laypeople to start CPR during the 2020 COVID-19 outbreak compared to 2019, while resuscitation attempts by BLS and ALS staff were not influenced by suspected/confirmed infection, even at univariable analysis. Our results suggest that basic and advanced treatment of OHCA is feasible and effective in selected patients also during COVID-19 pandemic.

## Supporting information

S1 Table Characteristics, resuscitation attempts and survival of suspected/confirmed COVID-19 patients. (DOCX)

S1 Fig Percentage of males, medical etiology, EMS-witnessed, bystander-witnessed, public location of OHCA, shockable presenting rhythm, bystander CPR, resuscitation attempted, ALS attempted and ROSC at hospital admission in overall 2020 patients (n = 694), overall 2019 patients (n = 520), 2020 patients excluding those with COVID-19 suspected or diagnosed (n = 555), 2020 patients under 65 years (n = 147) and 2019 patients under 65 years (n = 122). (TIF)

S1 Dataset (XLSX)
