# Supplementary material for: Treatment of out-of-hospital cardiac arrest in the COVID-19 era: A 100 days experience from the Lombardy region
Source: PLoS One. 2020 Oct 22;15(10):e0241028. doi: 10.1371/journal.pone.0241028 (PMC7580972; doi:10.1371/journal.pone.0241028)
Supplement: S1 Table — (DOCX) [file pone.0241028.s001.docx]

| ***Variable*** | ***N=139*** |
| --- | --- |
| **Males, n (%)** | 91 (65.5) |
| **Age, years [IQR]** | 76 [69-84] |
| **EMS arrival time, mins [IQR]** | 17 [12.2-23] |
| **Etiologies, n (%)** |  |
| *Medical* | 139 (100) |
| *Trauma* | 0 (0) |
| *Drowning* | 0 (0) |
| *Overdose* | 0 (0) |
| *Electrocution* | 0 (0) |
| *Asphyxial (external causes)* | 0 (0) |
| *Unknown* | 0 (0) |
| **OHCA locations, n (%)** |  |
| *Home* | 129 (92.8) |
| *Nursing residence* | 9 (6.5) |
| *Workplace* | 0 (0) |
| *Street* | 1 (0.7) |
| *Public building* | 0 (0) |
| *Sport* | 0 (0) |
| *Other* | 0 (0) |
| **Witnessed status, n (%)** |  |
| *Unwitnessed* | 41 (29.5) |
| *Bystander witnessed* | 63 (45.3) |
| *Witnessed by EMS* | 31 (22.8) |
| *Unknown* | 4 (2.9) |
| **Shockable presenting rhythm, n (%)** | 10 (7.6)  10 (10.5) ¥ |
| **Bystanders resuscitation** |  |
| Bystander CPR, n (%) | 17 (16.3)^  15 (22.4)† |
| AED use before EMS Arrival, n (%) | 1 (1) |
| **EMS Resuscitation** |  |
| Resuscitation attempted, n (%) | 96 (69.1) |
| Cause for not resuscitation, n (%) |  |
| *obviously dead* | 32 (23) |
| *considered futile* | 11 (7.9) |
| ALS attempted, n (%) | 36 (25.9)  36 (37.5)^¥^ |
| Mechanical compression, n (%) | 2 (5.6)* |
| Epinephrine, mg [IQR] | 3.5 [2-5]* |
| Amiodarone, n (%) | 3 (8.3)* |
| Shock delivered (mean±SD) | 0.8±1.7* |
| Resuscitation duration median^¶^ [IQR] | 43.6 [31.4-54.4] |
| **Outcome** |  |
| ROSC at hospital admission, n (%) | 9 (10.1) ^¥^  9 (26.5)* |
| IQR: 25-75 percentile range  EMS: Emergency Medical System  OHCA: out-of-hospital cardiac arrest  CPR: cardiopulmonary resuscitation  ROSC: return of spontaneous circulation  ¥ considering only patients with resuscitation attempted  ^ excluding EMS-witnessed patients  † excluding EMS-witnessed patients and considering only patients with resuscitation attempted  * considering only patients with ALS attempted  ¶ intended as the time from EMS arrival to the end of resuscitation | |
