# Supplementary figures and images for: Treatment of out-of-hospital cardiac arrest in the COVID-19 era: A 100 days experience from the Lombardy region
Source: PLoS One. 2020 Oct 22;15(10):e0241028. doi: 10.1371/journal.pone.0241028 (PMC7580972; doi:10.1371/journal.pone.0241028)

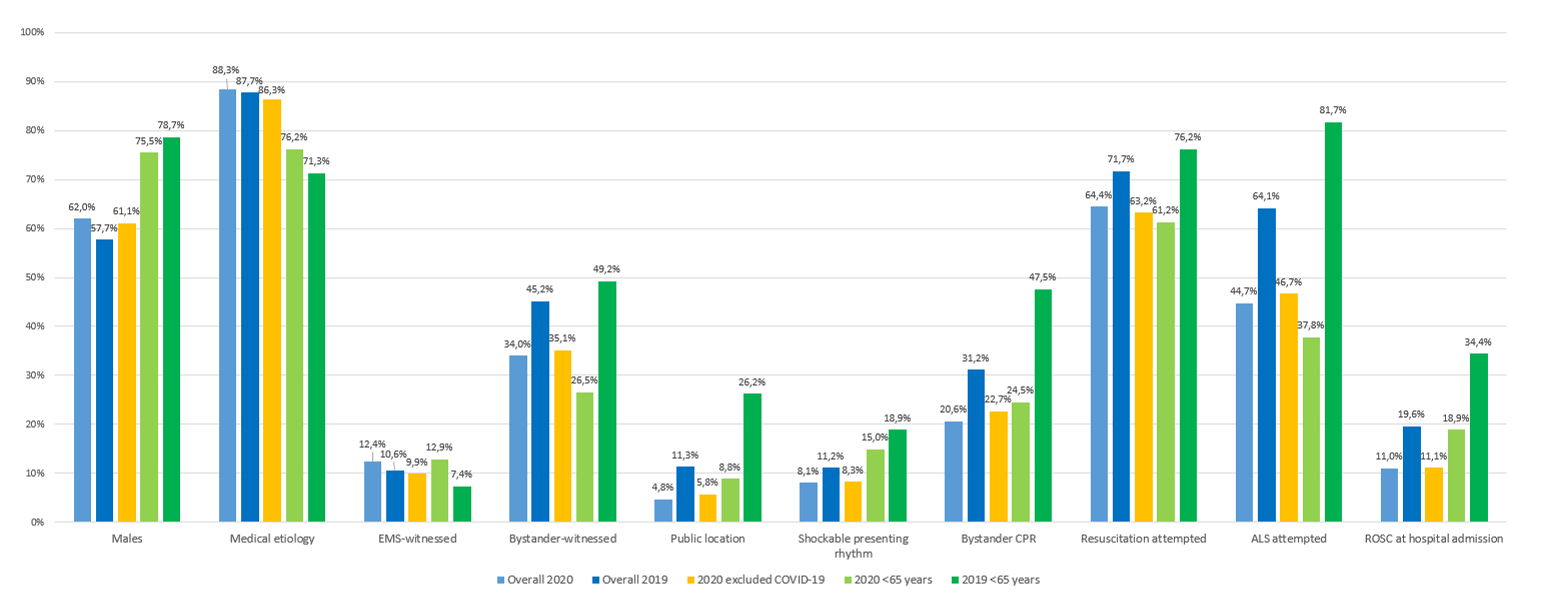

Supplement: S1 Fig — (TIF) [file pone.0241028.s002.tif]
